# The association between generative AI acceptance and college students’ career preparation: the serial mediating role of AI trust and career confidence and the moderating role of intrinsic motivation

**DOI:** 10.3389/fpsyg.2026.1954368

**Published:** 2026-10-08

**Authors:** Juan Jiang, Shuang Liang, Jiachun Chen

**Affiliations:** Zhongshan Institute, University of Electronic Science and Technology of China, Zhongshan, China

**Keywords:** AI trust, career confidence, career preparation, generative AI acceptance, intrinsic motivation

## Abstract

Against the backdrop that artificial intelligence (AI) is reshaping the labor market and occupational ecosystem, how college students build career preparation amid technological uncertainty has become a pressing question. Integrating the Unified Theory of Acceptance and Use of Technology (UTAUT) with Social Cognitive Career Theory (SCCT), this study constructs a research model in which AI acceptance is associated with career goal setting through AI trust and career confidence, and it examines the moderating role of intrinsic motivation. This study surveys undergraduate students in Chinese universities, obtaining 539 valid responses. The results show that (1) both performance expectancy and effort expectancy are positively associated with AI trust, with a stronger association for performance expectancy; (2) AI trust is positively associated with career confidence; (3) career confidence is positively associated with career goal setting; (4) AI trust and career confidence play a serial mediating role between performance expectancy, effort expectancy and career goal setting; (5) intrinsic motivation positively moderates the relationship between AI trust and career confidence, such that this association is stronger at higher levels of intrinsic motivation. These findings reveal how AI acceptance relates to college students’ career preparation and offer evidence for universities to implement AI literacy education and career counseling.

## 1 Introduction

The rapid development of Generative Artificial Intelligence (Gen-AI) fundamentally transforms skill requirements and career development within the global labor market (Deng and Sun, 2026; Ge et al., 2025). The World Economic Forum predicts that AI will create approximately 170 million new jobs and replace around 92 million existing positions by 2030. Nevertheless, alongside the job creation brought by AI, the problem of skill mismatch cannot be ignored. Newly created jobs are heavily concentrated in digitally intensive areas such as data analysis, AI development and human–AI collaboration, and they set high standards for practitioners’ technical literacy and interdisciplinary integration capabilities (Acemoglu and Restrepo, 2020; Zirar et al., 2023). Positions vulnerable to replacement mainly involve routine and repetitive basic work (Frey and Osborne, 2017), which serves as the traditional initial employment destination for numerous university graduates. The coexistence of high entry barriers for high-end jobs and continuous shrinkage of basic posts exposes contemporary college students to severe career uncertainty. More importantly, AI technologies also drive shifts in career patterns (Braganza et al., 2021; Brougham and Haar, 2018; Chowdhury et al., 2022). The logic of individual career development is shifting from relying on organizations for stable employment security to actively building sustainable adaptability, and from passively accepting assigned career roles to self-directed career path planning (He et al., 2023; Kong et al., 2021; Voigt and Strauss, 2024). Therefore, exploring individuals’ subjective perceptions of AI technological changes and their implications for career development become a core starting point to understand the logic of career construction in the new era (Kong et al., 2023; Presbitero and Teng-Calleja, 2023).

College students are facing the impacts of this AI transformation on career development, and their career development bears distinctive features. Unlike employed workers, they have not yet taken up specific occupational positions, so the formation of their career confidence and career goal setting relies more on imaginative construction than on realistic evaluation (Chen et al., 2025). In addition, college students are rapidly increasing their exposure to and reliance on AI (Pham Thi, 2026). At present, Gen-AI has widely penetrated their daily learning scenarios including thesis writing, programming assistance and information retrieval (Yilmaz et al., 2024). Nevertheless, existing theories fail to explain how college students’ acceptance of Gen-AI relates to their career preparation. Although classic theories in the technology acceptance field can explain why and how college students adopt AI, their outcome variables generally refer to behavioral intention and technology adoption rate (Tamilmani et al., 2021), and they seldom extend to occupational psychology. Meanwhile, literature within the career development field has examined relationships between AI perceptions and career outcomes, yet few studies directly incorporate antecedent variables of technology acceptance into analytical frameworks. The insufficient connection between research from the technology perspective and the career perspective creates a critical theoretical gap in studies concerning college students’ career development in the AI era.

Bearing on this gap, existing studies have accumulated preliminary evidence from two directions, yet an integrated explanatory framework has not been established. Research on employed workers indicates that AI cognition is double-edged: AI trust and AI awareness can enhance career sustainability, career competency, and innovative behavior, whereas AI awareness may also trigger adverse outcomes through emotional exhaustion (Kong et al., 2021, 2023; Liang et al., 2022). These conclusions, however, are drawn from samples of employed workers and cannot be directly applied to college students in the career exploration stage. Recent research has therefore turned to students, linking AI trust, awareness, and literacy to outcomes such as protean career orientation, employability, career expectations, career decision-making difficulties, and job-seeking anxiety, typically via a single mediator such as self-efficacy or intrinsic motivation (Ge et al., 2025; Ho and Le, 2026; Jiang and Chen, 2026; Li R. et al., 2025; Zhang et al., 2026). Despite these advances, three limitations remain unresolved. First, most existing research centers on such distal outcome constructs without examining college students’ career preparation itself—how AI relates to career confidence and career goal setting. Second, most studies adopt only a single mediator, overlooking the multi-step transmission path from technological cognition to career outcomes. Third, limited attention has been paid to the boundary conditions governing how technological attitudes transform into career outcomes. Accordingly, the current literature lacks an integrated theoretical model that incorporates antecedents of technology acceptance, reveals serial mediating mechanisms, and examines moderating boundary conditions simultaneously.

Based on the above theoretical integration, this study proposes and tests a research model to address three progressive research questions. At the first level (main effects): Do college students’ performance expectancy and effort expectancy positively predict their AI trust? Is AI trust positively associated with career confidence? Is career confidence positively associated with career goal setting? At the second level (serial mediation mechanism): Are performance expectancy and effort expectancy indirectly associated with career goal setting through the serial path of AI trust and career confidence? At the third level (moderating boundary condition): Does intrinsic motivation moderate the relationship between AI trust and career confidence, meaning that this association varies significantly with different levels of individual motivation? To answer these questions, this study selects 539 undergraduate students from Chinese universities as samples, collects data via questionnaire surveys, and applies hierarchical regression analysis and bootstrap tests for serial mediation effects to empirically verify the hypothesized model.

We expect this study to make the following contributions. First, this study expands the theoretical boundary of UTAUT. It links performance expectancy and effort expectancy to career goal setting via the serial path of AI trust and career confidence. This extension shifts the outcome variables of UTAUT from conventional technology usage behaviors to career psychological outcomes and extends the model beyond its long-standing confinement to behavioral intention. Second, this study enriches the theoretical connotation of SCCT within the digital occupational ecosystem. It incorporates AI trust into the antecedent framework of SCCT as environmental support on the technological dimension and addresses the theory’s insufficient attention to perceptions of the technological environment. Third, this paper identifies individual boundary conditions for the transformation from technological trust to career confidence. By testing the moderating effect of intrinsic motivation, it explains why the same level of AI trust is differentially associated with career confidence among different students. Drawing on Self-Determination Theory, the findings reveal the boundary conditions of individual motivational differences in the transformation from technology trust to career psychology, and provide empirical evidence for research on individual differences in career psychology amid the AI era. Fourth, the conclusions offer references for universities to develop tiered AI literacy programs and differentiated career guidance strategies. On one hand, identifying the antecedents of technology acceptance enables universities to advance AI education beyond basic tool operation toward value recognition. On the other hand, revealing the moderating role of intrinsic motivation helps educators implement tiered interventions targeting students with varying motivation levels.

## 2 Theoretical background and research hypotheses

### 2.1 Theoretical background

This study integrates UTAUT and SCCT to develop an analytical model suitable for understanding college students’ career preparation in the AI era. The rationale for this integration lies in the respective strengths of the two theories: UTAUT effectively explains why individuals accept technology (antecedents of technological attitudes), whereas SCCT elucidates how perceptions of the environment shape career outcomes. Logically, they jointly provide an integrated framework linking technological antecedents, technological attitudes, and subsequent career outcomes.

#### 2.1.1 Unified theory of acceptance and use of technology (UTAUT)

UTAUT integrates eight classic technology acceptance models (including TRA, TAM, MM, TPB, C-TAM-TPB, MPCU, IDT and SCT) through meta-analysis and proposes four core constructs that shape technology acceptance behavior (Venkatesh et al., 2003). Performance expectancy reflects the extent to which individuals believe technology can improve their job performance, and effort expectancy represents the extent to which individuals perceive technology as easy to use. Social influence refers to the degree to which important others think individuals should use technology, and facilitating conditions capture the extent to which individuals believe organizational and technical infrastructure supports technology adoption. Numerous empirical studies have verified that among the four core constructs, performance expectancy and effort expectancy serve as the most robust predictors of behavioral intention (Tamilmani et al., 2021).

The applicability of UTAUT has received preliminary verification in the context of AI technology. For instance, Yilmaz et al. (2024) developed an acceptance scale specifically for Generative AI based on the UTAUT framework. The scale covers four dimensions: performance expectancy, effort expectancy, social influence and facilitating conditions, and its reliability and validity have been verified using a sample of Turkish college students. Within higher education, multiple studies have found that performance expectancy and effort expectancy act as major driving forces for college students to adopt AI learning tools (Chatterjee and Bhattacharjee, 2020). Nevertheless, outcome variables in existing UTAUT research are generally limited to behavioral intention and use behavior. Few studies explore how technology acceptance further affects psychological outcomes outside the technological domain, such as career development. This limitation means that UTAUT can explain whether students are willing to use AI, yet it cannot answer the more practically meaningful educational question of whether and how the willingness to use AI influences their career development.

Among the four constructs of UTAUT, this study deliberately focuses on performance expectancy and effort expectancy while excluding social influence and facilitating conditions. This choice rests on four considerations. First, the theoretical aim of this study is to examine how individuals’ functional perceptions of generative AI—namely, its usefulness and ease of use—are translated into trust and, subsequently, into career-related psychological outcomes; performance expectancy and effort expectancy are the two core drivers of technology-related attitudes in UTAUT (Venkatesh et al., 2003) and are the most consistently supported predictors of AI acceptance among students (Tamilmani et al., 2021). Second, social influence reflects the normative expectations of important others; because college students’ use of generative AI tools is largely voluntary and self-initiated rather than mandated by an organization, the role of social influence is expected to be comparatively limited in this context. Third, facilitating conditions capture organizational and infrastructural support (e.g., technical infrastructure and assistance); for students who typically access generative AI applications through their own devices and free services, this dimension has limited applicability, and it has been found to play a weaker role in voluntary use contexts (Venkatesh et al., 2003). Fourth, the study aims to delineate a mechanism chain from technology perceptions to career psychology; including social-contextual dimensions would introduce constructs at a different level of analysis that lie beyond the scope of the proposed model. Accordingly, we focus on performance expectancy and effort expectancy as the technology-related antecedents.

#### 2.1.2 Social cognitive career theory

Social Cognitive Career Theory (SCCT) stands as one of the most influential theories in vocational psychology, and it is rooted in Bandura’s (1986) social cognitive theory. The core argument of SCCT holds that career development is jointly shaped by three social cognitive mechanisms: self-efficacy beliefs (individuals’ beliefs in their capability to complete specific tasks), outcome expectations (individuals’ imaginations of possible consequences stemming from certain behaviors), and career goals (individuals’ intentions to formulate and sustain specific career action plans) (Lent et al., 1994). SCCT further proposes that self-efficacy and outcome expectations originate from four types of learning experiences: performance accomplishments, vicarious learning, social persuasion, and physiological and affective states (Lent and Brown, 2019).

The key mechanism of SCCT indicates that environmental support boosts self-efficacy, self-efficacy facilitates career goals, and such progress ultimately leads to career actions (Lent and Brown, 2013). This mechanism has been validated in numerous empirical studies. For example, a longitudinal study conducted by Lent et al. (2016) found that social support for college students majoring in STEM significantly predicts their academic self-efficacy, which further promotes the formulation and persistence of academic goals. A meta-analysis by Rudolph et al. (2017) further confirmed stable positive relationships between dimensions of career adaptability (including career confidence) and adaptive outcomes (including progress toward career goals). Nevertheless, traditional SCCT mainly operationalizes environmental support as social factors such as teacher support, peer encouragement and family expectations, while paying limited attention to the technological environment. Especially against the backdrop where AI is reshaping the occupational ecosystem, it remains a theoretically worthy question to explore whether positive perceptions of AI technology can constitute a new form of environmental support.

#### 2.1.3 Logic and framework of theoretical integration

We propose an integrated framework of technology acceptance antecedents– perceived environmental support – career self-efficacy – career goals. First, performance expectancy and effort expectancy from UTAUT explain why college students develop positive attitudes toward AI. When students perceive AI as useful and easy to use, they are more likely to regard AI as a reliable auxiliary tool, namely forming AI trust. Second, AI trust is conceptualized as an environmental support element within the SCCT framework. Positive perceptions of AI technology indicate that students perceive a technological environment conducive to their development, and such environmental perception can strengthen their sense of control over their professional future (career confidence). Finally, in line with the core pathway of SCCT, career confidence promotes career goal setting. Throughout this study, career confidence is treated as the manifestation of career self-efficacy in the career preparation context.

It should be noted that career preparation is a multifaceted construct encompassing cognitive, behavioral, and contextual components (e.g., career decision-making, information exploration, and career engagement). In this study, we focus on two components of career preparation that are most directly linked to the mechanism examined here—career confidence and career goal setting. Following the CEDS framework (McCowan et al., 2024), career confidence is classified as an attitudinal component of career preparedness, whereas career goal setting is classified as a behavioral component. Throughout the manuscript, the term “career preparation” refers specifically to these two constructs and does not imply that they represent the entirety of career preparation.

### 2.2 Research hypotheses

#### 2.2.1 Performance expectancy, effort expectancy, and AI trust

As UTAUT’s core theoretical proposition, performance expectancy and effort expectancy are the two most important drivers of behavioral intention (Venkatesh et al., 2003). The proposition rests on two different cognitive evaluative processes. Performance expectancy reflects instrumental cognition: positive utility evaluations occur when people believe a technology helps them achieve goals and enhance performance. Effort expectancy reflects ease-of-use cognition: when perceived complexity and learning costs are low, the psychological barrier to adoption decreases and the evaluation involves self-efficacy judgment. Davis (1989) first distinguished perceived usefulness and perceived ease of use in the TAM; this distinction was later integrated into UTAUT by Venkatesh et al. (2003). Both constructs consistently shape behavioral intention, as documented by Tamilmani et al. (2021). Moreover, the effect size of performance expectancy is typically larger than the effect size of effort expectancy, showing that instrumental value judgment plays a decisive role in the formation of technology attitudes.

In general information technology usage scenarios, performance expectancy and effort expectancy are typically associated with users’ attitudes or willingness to use the technology. However, in the specific context of AI technology, trust is more crucial than general usage attitudes (Chowdhury et al., 2022; Kong et al., 2023). The core reason for this difference lies in the unique characteristics of AI compared to traditional information technology: AI possesses the ability to operate autonomously (Parent-Rocheleau and Parker, 2022), and can autonomously make decisions and generate content (Einola and Khoreva, 2023; Zhao et al., 2025); moreover, AI exhibits black-box characteristics (Floridi et al., 2018; Zirar et al., 2023), whose internal mechanisms ordinary users cannot understand (Bankins and Formosa, 2023; Qin et al., 2025). Users cannot build confidence in using AI by understanding its operating principles; they can only rely on trust to alleviate their inner doubts (Huo et al., 2025). In addition, generative AI iterates extremely rapidly (Luo et al., 2025; Raisch and Krakowski, 2021). Long-term use therefore cannot rest on a single adoption decision; stable and continuous trust is essential. Therefore, in research on user acceptance of AI, trust has a stronger explanatory power than attitudes and willingness to use, and is a more important proximate outcome variable (Glikson and Woolley, 2020).

Performance expectancy fosters trust through a value-affirmation pathway. When students believe that generative AI can efficiently assist in various academic tasks such as essay writing and information retrieval, and thus develop high performance expectations, they will view AI as a valuable tool (Ho and Le, 2026). This positive tool evaluation enhances individuals’ trust in the AI’s capabilities and the reliability of its output. In contrast, effort expectancy drives trust development through a psychological accessibility pathway. If students perceive AI as having a user-friendly interface, simple operation, and requiring no specialized skills to use, they will have high effort expectations. This shortens the psychological distance between people and AI, reduces cognitive resistance (Wang and Gao, 2025), and makes them more willing to apply AI in daily learning and problem-solving, gradually building corresponding trust.

Existing research has confirmed the association between performance expectancy and effort expectancy and positive attitudes towards AI and trust in AI. For instance, Yilmaz et al. (2024) showed that both performance expectancy and effort expectancy significantly predict college students’ willingness to use generative AI, with performance expectancy having higher predictive power. Duong et al. (2023), using a sample of 1,389 college students in Vietnam, further found that effort expectations are positively associated with the actual use of ChatGPT through a serial mediation path of performance expectations and behavioral intentions, confirming the dual-driven model of performance expectancy and effort expectancy in college students’ AI adoption. In the organizational context, Kong et al. (2023) found that employees’ positive evaluation of AI capabilities is the cognitive basis for their development of trust in AI. Thus, the process by which university students develop trust in AI through firsthand experience relies primarily on their initial functional perception of AI—that is, whether AI is useful to them and how easy it is to use. Therefore, we propose:

Hypothesis 1: Performance expectancy is positively associated with AI trust.

Hypothesis 2: Effort expectancy is positively associated with AI trust.

#### 2.2.2 AI trust and career confidence

SCCT situates the development of self-efficacy beliefs within a social cognitive framework and posits that individuals’ beliefs in their own capabilities stem from learning experiences (Lent et al., 1994). Nevertheless, SCCT further emphasizes that these learning experiences do not operate in a vacuum. As contextual factors, environmental supports and environmental barriers directly shape self-efficacy (Brown and Lent, 2019). Even if individuals have accumulated substantial learning experiences, the development of self-efficacy can still be inhibited when the surrounding environment conveys negative and obstructive signals. Conversely, a supportive environment can directly foster self-efficacy.

To further clarify this theoretical positioning, three points deserve emphasis. First, in SCCT, environmental supports are inherently conceptualized as individuals’ perceptions of the facilitative conditions in their environment rather than as purely objective contextual features (Lent et al., 1994; Lent and Brown, 1996). Family support, institutional resources, and social encouragement is associated with career development precisely through the individual’s perceived availability of such support. Perceived in this way, AI trust—as a judgment about whether the technological environment is capable, reliable, and beneficial—operates at the same level of analysis as other perceived environmental supports. Second, AI trust is distinct from the cognitive person variables of SCCT in terms of its object of evaluation: career self-efficacy concerns beliefs about one’s own capabilities, and outcome expectations concern the anticipated consequences of one’s own behaviors, whereas AI trust is an evaluation of an external, contextual factor (i.e., AI technology as part of the future occupational environment). Accordingly, AI trust occupies the position of an environmental support in the SCCT pathway, predicting career self-efficacy, rather than duplicating the role of self-efficacy or outcome expectations. Third, the technological environment has become an increasingly relevant background condition for career development in the digital era, and recent SCCT applications have begun to incorporate technology-related contextual factors as environmental factors associated with career outcomes (Brown and Lent, 2019). On these grounds, conceptualizing AI trust as a technology-related environmental support is consistent with, and extends, the SCCT framework rather than departing from it.

When applying this theoretical mechanism to career contexts in the AI era, AI trust can be conceptualized as a special form of technology-related contextual support perception. The first aspect lies in the signaling mechanism of AI trust as environmental cognition. When college students trust AI, it represents more than merely a positive attitude toward a technological tool. More importantly, such trust conveys cognitive signals regarding what the future occupational landscape will look like (Imjai et al., 2025). Students who trust AI tend to believe that AI technology will continue to evolve and exert positive influences in society, and that AI will not fully replace human workers but reshape workflows through human–machine collaboration (Wang et al., 2025). Furthermore, they hold the view that proficiency in collaborating with AI will become a crucial component of future career competitiveness (opportunity expectation cognition). These cognitions jointly form a psychological framework of technological environmental support. When students interpret their own career prospects within this framework, they perceive less uncertainty and an enhanced sense of control, which in turn fosters higher career confidence (Zhang et al., 2026).

In addition, the effect of AI trust on career confidence should be understood in conjunction with the distinct implications of AI technology for college students. Unlike employed workers, college students have not yet taken up specific occupational positions. Their career confidence is built more on the imaginative construction of what they will be capable of doing in the future than on realistic evaluation of what they are currently doing. One of the psychological challenges brought by the rapid penetration of AI among college students is AI anxiety (Li J. M. et al., 2025; Papagiannidis et al., 2025), which reflects worries and fears that AI may displace future employment opportunities (Wang and Wang, 2022). Under such anxious circumstances, trust in AI serves a vital buffering function. Students who trust AI do not regard it as an occupational replacement threat but as a career enabler. Such cognitive restructuring directly safeguards and even strengthens their career confidence. Thus, we propose:

Hypothesis 3: AI trust is positively associated with career confidence.

#### 2.2.3 Career confidence and career goal setting

One of the core propositions of SCCT is that self-efficacy beliefs directly drive goal-setting behaviors (Lent et al., 1994). Grounded in Bandura’s (1986) social cognitive theory, this proposition rests on the assumption of human agency: individuals actively shape their development through self-referent thought. Self-efficacy influences goal setting through three functions (Bandura, 1977, 1991): shaping the selection of higher and more challenging goals, motivating effort and resource devotion, and sustaining commitment and persistence in the face of obstacles and setbacks.

Within the specific context of career development, SCCT distinguishes two forms of goal setting: choice goals (decisions to enter a particular occupational field) and performance goals (striving to attain specific standards within the chosen field) (Lent and Brown, 1996, 2019). For college students who have not yet entered the workforce, career goal setting mainly manifests as the former. They need to make preliminary choices among numerous potential career paths and formulate corresponding educational and developmental plans (Lent et al., 1994). This process requires students to integrate two types of information: self-related information (“what am I capable of and good at”) and external environmental information (“what career opportunities are available and what requirements these opportunities entail”). Career confidence reflects students’ positive evaluations on the self-information dimension. Such positive evaluations embolden students to establish concrete, clear career goals and hold reasonable expectations of goal attainment (McCowan et al., 2024).

Empirical evidence strongly supports the relationship between self-efficacy and goal setting. In a meta-analysis covering numerous independent studies, Rudolph et al. (2017) identified robust positive effects between dimensions of career adaptability and adaptive outcomes, among which the correlation between career confidence and career planning stands out remarkably. Another meta-analysis conducted by Sheu et al. (2018) examining the core propositions of SCCT also revealed a significant direct effect of self-efficacy on career goals. Among college student samples, the CEDS scale developed by McCowan et al. (2024) explicitly classifies confidence as an attitudinal component and goal setting as a behavioral component of career preparedness, thereby drawing a clear conceptual distinction between the two. The two components are closely correlated empirically, consistent with the theoretical position of attitudinal confidence as an antecedent of goal-setting behavior. A study of higher education students by Jackson and Tomlinson (2019) additionally found that value-oriented career attitudes effectively predict students’ proactive career planning behaviors. This finding indicates that career goal setting among contemporary college students has transcended conventional externally driven models and relies more heavily on internal career values and self-efficacy.

Notably, amid the widespread penetration of AI technology, the relationship between career confidence and career goal setting may gain an additional reinforcing association. For students who hold confidence in their professional futures, the emergence of AI technology will not undermine their career goals (Ho and Le, 2026). Instead, it may motivate them to incorporate the new dimension of human–machine collaboration into goal formulation. For instance, they may set goals to become individuals who can leverage AI effectively and adopt more proactive behaviors (Gerçek and Özveren, 2025). Accordingly, we propose:

Hypothesis 4: Career confidence is positively associated with career goal setting.

#### 2.2.4 The serial mediating role of AI trust and career confidence

The foregoing analyses elaborate on the theoretical associations between adjacent variables within the model. Nevertheless, the theoretical value of these linkages resides not merely in their individual validity, but in their collective formation of an overarching mechanism extending from technological perceptions to career outcomes. Building on these arguments, we propose two serial mediation pathways: performance expectancy and effort expectancy are expected to relate to career goal setting indirectly through the serial mediation of AI trust and career confidence.

When college students believe that AI can help them achieve better outcomes in their studies (performance expectancy), they regard AI as a reliable auxiliary resource (AI trust); such trust signals a favorable technological environment, enhancing their career confidence, which in turn motivates them to formulate clear career goals. Effort expectancy initiates the same serial process through perceptions of ease of use: students who find AI easy to master experience lower psychological barriers, develop trust in AI, and thereby progress to career goal setting through the same chain. Therefore, we propose that:

Hypothesis 5: Performance expectancy is indirectly associated with career goal setting through the serial mediation of AI trust and career confidence.

Hypothesis 6: Effort expectancy is indirectly associated with career goal setting through the serial mediation of AI trust and career confidence.

#### 2.2.5 The moderating role of intrinsic motivation

Intrinsic motivation is a tendency to engage in an activity for its own sake because one is interested in or satisfied with the activity (Deci and Ryan, 2000). According to SDT, intrinsic motivation arises from the satisfaction of three basic psychological needs—competence, autonomy, and relatedness (Deci and Ryan, 2008). Intrinsically motivated individuals are therefore more likely to actively process information and change their behavior when encountering external information, for example, AI trust information (Jiang and Chen, 2026).

In the interdisciplinary context of AI and career development, the mechanism through which intrinsic motivation moderates the relationship between AI trust and career confidence can be analyzed via two complementary pathways: depth of information processing and proactivity of behavioral transformation. From the perspective of depth of information processing, students with high intrinsic motivation hold attitudes toward AI technology that go beyond mere personal belief in AI and entail in-depth cognitive engagement (Jiang and Chen, 2026). They proactively reflect on how AI can be integrated with their majors and career interests, how AI may help them compensate for gaps in knowledge and skills, and how they can achieve higher career goals with AI assistance (Gerçek and Özveren, 2025). Such thorough information processing grounds AI trust from an abstract attitudinal level down to the concrete level of vocational self-concept, enabling it to translate into career confidence more effectively (Li, Ouyang, et al., 2025).

From the perspective of the behavioral proactivity pathway, students with high intrinsic motivation are more inclined to translate positive attitudes into concrete actions. Within the specific context of AI trust, this means they not only trust AI but also actively leverage AI tools for career information searching (e.g., retrieving industry trends and job requirements via AI), skill learning (e.g., practicing programming or design with AI support), and career exploration (e.g., conducting mock interviews with AI or evaluating the fit of different career paths) (Pham Thi, 2026; Testa et al., 2026). These tangible actions transform abstract AI trust into concrete career preparation experiences. According to SCCT, such experiences—particularly learning experiences derived from performance accomplishments—serve as sources of self-efficacy (Lent et al., 2022). Therefore, high intrinsic motivation amplifies the positive association between AI trust and career confidence by activating the behavioral transformation mechanism. In contrast, even if students with low intrinsic motivation cognitively trust AI, they lack the internal drive to translate such trust into exploratory and preparatory behaviors, which weakens the empowering association of AI trust. Accordingly, we propose:

Hypothesis 7: Intrinsic motivation positively moderates the relationship between AI trust and career confidence, such that the positive association between AI trust and career confidence is stronger when intrinsic motivation is higher.

Our research model is presented in Figure 1.

**Figure 1:**
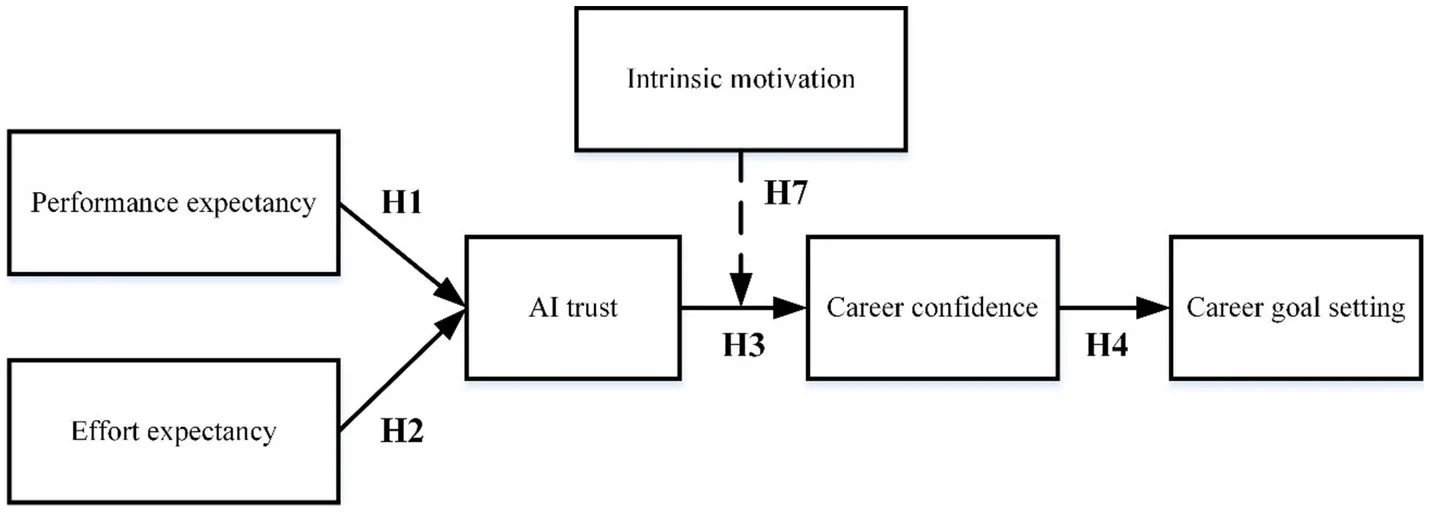
Research model. H5: Performance expectancy → AI trust → Career confidence → Career goal setting; H6: Effort expectancy → AI trust → Career confidence → Career goal setting; Solid lines indicate direct paths; Dashed lines represent moderation effects.

## 3 Methods

### 3.1 Sample and data sources

This study adopted a questionnaire survey method. Questionnaires were distributed to college students in Chinese universities via the online platform Credamo. Data collection was conducted in February 2026. A total of 580 questionnaires were distributed. After eliminating invalid responses such as those with excessively short completion time and patterned answering, 539 valid questionnaires were retained.

The sample included 336 female students (62.3%) and 203 male students (37.7%). In terms of academic year, first-year students accounted for 23.6%, second-year students 45.8%, third-year students 21.1%, and fourth-year students and above 9.6%. The average age was 20.04 years (SD = 1.31). Majors covered multiple disciplines including science and engineering, economics and management, and humanities.

Participants were recruited through the Credamo online survey platform, which maintains a large registered panel of respondents. A convenience sampling strategy was adopted: the survey link was distributed to registered panel members who met the inclusion criterion of being current full-time undergraduate students at Chinese universities, and each respondent was permitted to complete the questionnaire only once. Before answering, respondents were asked to confirm their enrollment status. The 539 valid responses were distributed across 30 provincial-level regions of China, with approximately two-thirds of the respondents (66.4%) located in Guangdong Province and the remainder spread across most other provinces and municipalities (e.g., Sichuan, Shandong, Fujian, and Jiangsu). To protect anonymity and encourage candid responding, the questionnaire did not ask respondents to name their university; accordingly, the number and distribution of the participating universities cannot be reported, and the provincial-level distribution above is the most detailed available description of the sample’s institutional coverage.

### 3.2 Measures

All scales used a 5-point Likert format (1 = strongly disagree, 5 = strongly agree). Chinese versions of all scales were developed through a translation and back-translation procedure.

The translation and adaptation process followed a rigorous procedure to ensure conceptual equivalence for Chinese university students. First, all English instruments were independently translated into Chinese by two bilingual researchers with expertise in psychology and management, and discrepancies were resolved through discussion. Second, following the standard translation–back-translation procedures recommended in the cross-cultural literature (Brislin, 1970), the Chinese versions were back-translated into English by two independent bilingual translators who were blind to the original instruments; the back-translations were then compared with the original English versions, and any semantic inconsistencies were corrected to ensure conceptual equivalence. Third, because several scales were originally developed in employee or organizational contexts (e.g., the AI trust scale; Chowdhury et al., 2022), the item wording was carefully adapted to the university-student context (e.g., references to organizational settings were rephrased toward students’ daily academic and career-development contexts), while preserving the original meaning of each item. Finally, the psychometric properties of the adapted scales were evaluated in the main sample: the confirmatory factor analysis reported in Section 4.1 yielded significant factor loadings for all items, and the reliability, composite reliability, and AVE of each scale were assessed (see Table 1). These steps jointly support the conceptual equivalence and validity of the translated and context-adapted instruments for Chinese university students.

**Table 1:** Reliability and validity.

Variables	α	CR	AVE	HTMT					
				1	2	3	4	5	6
1. Performance expectancy	0.853	0.882	0.517	—					
2. Effort expectancy	0.812	0.861	0.556	0.812	—				
3. AI trust	0.879	0.897	0.447	0.723	0.617	—			
4. Intrinsic motivation	0.847	0.872	0.535	0.688	0.574	0.622	—		
5. Career confidence	0.768	0.852	0.660	0.505	0.630	0.510	0.537	—	
6. Career goal setting	0.736	0.846	0.648	0.538	0.622	0.555	0.573	0.880	—

α = Cronbach’s alpha; CR = Composite Reliability; AVE = Average Variance Extracted. HTMT = Heterotrait-Monotrait ratio.

*Performance expectancy* was measured using the seven-item scale from the Generative AI Acceptance Scale developed by Yilmaz et al. (2024). A sample item is “I find generative AI applications useful in my daily life.”

*Effort expectancy* was measured using the five-item subscale from the Generative AI Acceptance Scale developed by Yilmaz et al. (2024). A sample item is “Learning how to use generative AI applications is easy for me.”

*AI trust* was measured using the 11-item AI trust scale adopted by Kong et al. (2023). A sample item is “I believe AI technology can facilitate routine and trivial tasks through automation.”

*Intrinsic motivation* was measured using the six-item intrinsic motivation scale developed by Liang et al. (2022). A sample item is “To overcome the uncertainty of AI, I want to know how well I can perform in my future job.”

*Career confidence* was measured using the confidence subscale from the Career Education and Development Scale developed by McCowan et al. (2024), which consists of three items. A sample item is “I feel confident that I have a good idea of what career direction(s) or pathways I want to take.”

*Career goal setting* was measured using the goal-setting subscale from the Career Education and Development Scale developed by McCowan et al. (2024), which contains three items. A sample item is “I have developed a career plan for myself.”

*Gender* and *age* were included as control variables. Gender was operationalized as a dummy variable, coded 1 for male and 0 for female.

### 3.3 Data analysis strategy

Statistical analyses were conducted using R 4.5. First, Harman’s single-factor test was performed to assess common method bias, and Cronbach’s α coefficients were calculated to examine scale reliability. Second, the validity of the measurement model was evaluated, with average variance extracted (AVE), composite reliability (CR), and HTMT ratios reported for each scale. Third, descriptive statistics and Pearson correlation analyses were carried out. Next, hierarchical regression analysis was adopted to test the main effects. Gender and age were included as control variables, and complete regression equations were established with AI trust, career confidence, and career goal setting as dependent variables. Bootstrap resampling with 5,000 iterations and 95% percentile confidence intervals was then used to examine the serial mediation effects, with performance expectancy and effort expectancy entered simultaneously as predictors and AI trust and career confidence as serial mediators. Finally, an interaction term was constructed to test the moderating effect of intrinsic motivation on the association between AI trust and career confidence, followed by simple slope analysis.

Regarding the analytical strategy, we employed hierarchical regression analysis with bootstrap resampling rather than a full structural equation model (SEM), for three reasons. First, the hypothesized structure—main effects, serial mediation, and moderation—maps directly onto the regression framework, which, combined with bootstrap confidence intervals, is a well-established and transparent approach (Hayes, 2018). Second, measurement error was addressed at the measurement level through the six-factor confirmatory factor analysis reported in Section 4.1. Third, performance expectancy and effort expectancy are moderately correlated (r = 0.67, see Table 2); multicollinearity of this kind affects coefficient estimation in regression-based and latent-variable approaches alike, and we therefore do not claim that regression is immune to it. In the present data, however, the degree of collinearity is modest: the variance inflation factors (VIFs) for the two predictors in the regression models were approximately 1.8, far below conventional thresholds (e.g., VIF < 5), indicating that their standard errors were inflated by at most about 36%. Because the hypotheses concern whether each technology-perception antecedent is positively associated with AI trust and whether these associations propagate through the hypothesized mediation chain—rather than the precise decomposition of their relative contributions—the regression framework provides transparent, interpretable tests that map directly onto the hypothesized model, with measurement quality established separately through the six-factor CFA (Section 4.1). These considerations justify the use of regression-based procedures, which complement rather than substitute for the measurement model validation.

**Table 2:** Descriptive statistics and correlation matrix.

Variables	*M*	SD	1	2	3	4	5	6	7	8
1. Performance expectancy	3.74	0.57	1							
2. Effort expectancy	3.61	0.62	0.674***	1						
3. AI trust	3.58	0.60	0.609***	0.513***	1					
4. Intrinsic motivation	3.58	0.67	0.581***	0.469***	0.523***	1				
5. Career confidence	3.50	0.74	0.402***	0.490***	0.420***	0.425***	1			
6. Career goal setting	3.44	0.75	0.415***	0.470***	0.445***	0.444***	0.660***	1		
7. Gender	0.38	0.48	0.036	0.093*	0.049	0.061	0.178***	0.105*	1	
8. Age	20.04	1.31	0.005	0.057	0.137**	0.038	0.169***	0.093*	0.161**	1

*N* = 539; *p < 0.05, **p < 0.01, ***p < 0.001; Gender is a dummy variable (male = 1, female = 0).

## 4 Results

### 4.1 Common method bias, reliability, and validity

Harman’s single-factor test revealed that the first unrotated factor accounted for 33.56% of the total variance, which was below the critical threshold of 40% (Podsakoff et al., 2003). This indicated that common method bias in this study was within an acceptable range.

To further rule out common method bias, we supplemented the single-factor test with more rigorous procedures. First, we compared a single-factor model, in which all items loaded on one common factor, with the hypothesized six-factor measurement model in a confirmatory factor analysis (CFA). The single-factor model exhibited poor fit (CFI = 0.699, TLI = 0.680, RMSEA = 0.090, SRMR = 0.078), whereas the six-factor model fitted the data well (CFI = 0.910, TLI = 0.902, RMSEA = 0.050, SRMR = 0.046), and the chi-square difference was significant (Δχ2 = 1741.01, Δdf = 15, *p* < 0.001), indicating that a single method factor cannot adequately account for the observed relationships among the constructs. Second, we applied the common latent factor (CLF) approach by adding an orthogonal method factor to the measurement model. The average standardized loading on the method factor was small (mean |λ| = 0.169), and the loadings of the substantive factors remained essentially unchanged after controlling for the method factor (mean |Δloading| = 0.026; none exceeded 0.20), suggesting that method variance, although statistically detectable, had a limited impact on measurement. Together, these analyses provide convergent evidence that common method bias was unlikely to have materially influenced the results.

The results of reliability and validity analysis are presented in Table 1. The Cronbach’s α values of all scales ranged from 0.736 (career goal setting) to 0.879 (AI trust), all exceeding the acceptable threshold of 0.70, which demonstrated good internal consistency of the measurement instruments. Composite reliability (CR) values for each scale fell between 0.846 and 0.897, above the benchmark of 0.70. Average variance extracted (AVE) ranged from 0.447 (AI trust) to 0.660 (career confidence). The AVE values of performance expectancy, effort expectancy, intrinsic motivation, career confidence, and career goal setting met the recommended cutoff of 0.50 (Fornell and Larcker, 1981). The AVE of AI trust (0.447) was slightly below this threshold. Nevertheless, given its CR value of 0.897, the convergent validity can still be regarded as acceptable.

To provide a more complete assessment of the measurement model, we conducted a confirmatory factor analysis (CFA) of the hypothesized six-factor structure. The model showed satisfactory fit (χ2 = 1276.86, df = 545, CFI = 0.910, TLI = 0.902, RMSEA = 0.050, SRMR = 0.046). In addition, the standardized loadings of all items were examined: they ranged from 0.69 to 0.78 for performance expectancy, 0.72 to 0.79 for effort expectancy, 0.42 to 0.77 for AI trust, 0.70 to 0.80 for intrinsic motivation, 0.80 to 0.84 for career confidence, and 0.79 to 0.83 for career goal setting. Specifically, the loadings of the 11 AI trust items were 0.69 (AT1), 0.64 (AT2), 0.42 (AT3), 0.72 (AT4), 0.72 (AT5), 0.76 (AT6), 0.73 (AT7), 0.70 (AT8), 0.73 (AT9), 0.54 (AT10), and 0.77 (AT11). The complete list of measurement items and their standardized factor loadings is provided in the Appendix Table A1.

Although the AVE of AI trust (0.447) fell slightly below the conventional threshold of 0.50, several lines of evidence support its convergent validity. First, composite reliability (CR = 0.897) and Cronbach’s α (0.879) were well above the recommended benchmarks, and all factor loadings were statistically significant. Second, the AVE criterion was originally proposed by Fornell and Larcker (1981) as a suggested guideline rather than a strict cutoff; when composite reliability is high, an AVE in the range of 0.40–0.50 can still be regarded as acceptable evidence of convergent validity (Malhotra et al., 2006). Third, the AI trust scale used in this study was originally developed by Chowdhury et al. (2022) and subsequently adopted in AI-integrated organizational research (Kong et al., 2023). Within this framework, AI trust is conceptually defined as individuals’ positive perceptions of AI and its potential impacts on their roles and responsibilities (Chowdhury et al., 2022). The scale items map onto this definition: the first four items (AT1–AT4) capture confidence in AI’s capabilities and reliable operation, whereas the remaining items (AT5–AT11) capture positive attitudes toward AI adoption and expectations regarding its beneficial consequences for work and careers—precisely the ‘potential impacts’ component of the construct definition. Moreover, the scale demonstrated satisfactory convergent validity in its original development sample (AVE = 0.656), well above the 0.50 threshold. The comparatively lower AVE (0.447) in the present study is therefore more plausibly attributable to sample and contextual differences (e.g., Chinese university students versus UK creative-industry employees, translation and context adaptation) than to a defect in the measurement instrument itself. We therefore retained all 11 items to preserve the content validity of the construct.

Discriminant validity was assessed using the HTMT ratio (Henseler et al., 2015). Taking 0.90 as the cutoff criterion, all HTMT values between constructs were lower than 0.90 (see Table 1), indicating satisfactory discriminant validity across constructs.

It should be noted that the HTMT value between career confidence and career goal setting (0.880) approached the 0.90 criterion. This is expected given their conceptual proximity: both constructs originate from the same career development framework, and career confidence is theoretically the immediate antecedent of goal-setting behavior, occupying the attitudinal component that precedes the behavioral component of career preparedness (McCowan et al., 2024). The Fornell–Larcker criterion was nevertheless satisfied, as the square root of the AVE for career confidence (0.81) and for career goal setting (0.80) both exceeded the correlation between the two constructs (*r* = 0.66), supporting the empirical distinctiveness of career confidence and career goal setting.

### 4.2 Descriptive statistics and correlation analysis

Table 2 reports the means, standard deviations, and Pearson correlation coefficients of all variables. The mean values of the variables were moderately high (ranging from 3.44 to 3.74 on a 5-point scale), with standard deviations between 0.57 and 0.75, suggesting a relatively concentrated data distribution. Specifically, performance expectancy had the highest mean (*M* = 3.74, SD = 0.57), whereas career goal setting yielded the lowest mean (*M* = 3.44, SD = 0.75). In terms of correlation patterns, all six focal variables exhibited significant positive correlations (*p* < 0.001), offering preliminary support for subsequent hypothesis testing. Specifically, performance expectancy was significantly and positively correlated with AI trust (*r* = 0.609, *p* < 0.001), and effort expectancy was significantly and positively correlated with AI trust (*r* = 0.513, *p* < 0.001). This reveals that students’ perceptions of AI usefulness and ease of use are closely associated with their trust in AI. AI trust was significantly positively correlated with career confidence (*r* = 0.420, *p* < 0.001), indicating that students with greater trust in AI hold higher confidence in their career prospects. Career confidence showed a significant positive correlation with career goal setting (*r* = 0.660, *p* < 0.001), suggesting a strong linkage between career confidence and goal formulation.

### 4.3 Hierarchical regression and hypothesis testing

The results of regression model tests are shown in Table 3. With AI trust as the dependent variable (Model 1), performance expectancy (*b* = 0.514, *p* < 0.001) and effort expectancy (*b* = 0.172, *p* < 0.001) positively predicted AI trust, supporting Hypothesis 1 and Hypothesis 2. When career confidence served as the dependent variable, AI trust positively predicted career confidence in Model 2 (b = 0.496, *p* < 0.001), which confirmed Hypothesis 3. Regarding career goal setting as the dependent variable, career confidence positively predicted career goal setting in Model 5 (b = 0.672, *p* < 0.001), consistent with Hypothesis 4.

**Table 3:** Hierarchical regression analysis results.

Variables	Model 1 AI trust	Model 2 Career confidence	Model 3 Career confidence	Model 4 Career goal setting	Model 5 Career goal setting	Model 6 Career goal setting	Model 7 Career confidence
Performance expectancy	0.514***	—	0.063	0.245***	—	0.051	—
	(10.79)		(0.89)	(3.65)		(0.83)	
Effort expectancy	0.172***	—	0.408***	0.408***	—	0.134*	—
	(3.90)		(6.82)	(6.56)		(2.45)	
AI trust	—	0.496***	0.243***	—	—	0.180***	0.331***
		(10.32)	(4.20)			(3.49)	(6.03)
Career confidence	—	—	—	—	0.672***	0.540***	—
					(19.92)	(14.25)	
Intrinsic motivation	—	—	—	—	—	—	0.330***
							(6.72)
AI Trust × Intrinsic motivation	—	—	—	—	—	—	0.107*
							(2.06)
Age	0.057***	0.052*	0.059**	0.037	−0.010	−0.013	0.058**
	(3.69)	(2.31)	(2.78)	(1.68)	(−0.52)	(−0.69)	(2.71)
Gender (male)	−0.006	0.221***	0.183**	0.088	−0.017	−0.008	0.186**
	(−0.15)	(3.70)	(3.24)	(1.49)	(−0.32)	(−0.17)	(3.20)
*R* ^2^	0.405	0.209	0.307	0.247	0.436	0.482	0.312

*N* = 539; *p < 0.05, **p < 0.01, ***p < 0.001; Values in parentheses are *t*-values. Coefficients are unstandardized regression coefficients. Gender is a dummy variable (male = 1, female = 0).

To test the moderating effect of intrinsic motivation (Hypothesis 7), Model 7 took career confidence as the dependent variable. Centered AI trust, intrinsic motivation, their interaction term (AI trust × intrinsic motivation), together with control variables were entered into the model. The interaction term AI trust × intrinsic motivation was significant (*b* = 0.107, *p* = 0.040), which supported Hypothesis 7.

The serial mediation effects were examined via bootstrap resampling with 5,000 iterations, controlling for gender and age (see Table 4). Performance expectancy and effort expectancy were entered simultaneously as predictors in a single model, with AI trust and career confidence serving as mediators. The total effect of performance expectancy on career goal setting (0.245) decomposed into a direct effect (0.051) and a total indirect effect (0.193), the latter comprising the indirect effect through AI trust (0.092), through career confidence (0.034), and through the serial path AI trust → career confidence (0.067). The total effect of effort expectancy (0.408) likewise decomposed into a direct effect (0.134) and a total indirect effect (0.274), comprising the indirect effect through AI trust (0.031), through career confidence (0.221), and through the serial path (0.023). The serial indirect effects were significant (95% percentile CIs [0.023, 0.119] and [0.006, 0.047], respectively), supporting Hypotheses 5 and 6.

**Table 4:** Total, direct, and indirect effects for performance expectancy and effort expectancy.

**Path**	**Total effect**	**Direct effect**	**Indirect effect** **via AI trust**	**Indirect effect** **via career confidence**	**Serial indirect effect**	**Total indirect effect**
Performance expectancy → AI trust → Career confidence → Career goal setting	0.245	0.051	0.092 [0.033, 0.158]	0.034 [−0.052, 0.127]	0.067 [0.023, 0.119]	0.193 [0.102, 0.290]
Effort expectancy → AI trust → Career confidence → Career goal setting	0.408	0.134	0.031 [0.008, 0.064]	0.221 [0.149, 0.301]	0.023 [0.006, 0.047]	0.274 [0.196, 0.362]

*N* = 539. Performance expectancy and effort expectancy were entered simultaneously as predictors of career goal setting, with AI trust and career confidence as mediators; gender and age were controlled. Estimates are unstandardized OLS coefficients. Values in brackets are 95% percentile bootstrap confidence intervals (5,000 resamples) for the indirect effects. The total effect equals the direct effect plus the total indirect effect.

Simple slope analysis is illustrated in Figure 2. When intrinsic motivation was low (−1 SD), the association between AI trust and career confidence remained significant (simple slope = 0.260, SE = 0.060, *p* < 0.001). When intrinsic motivation was high (+1 SD), the association between AI trust and career confidence became stronger (simple slope = 0.403, SE = 0.070, *p* < 0.001). This finding indicates that the positive association between AI trust and career confidence was stronger at higher levels of intrinsic motivation.

**Figure 2:**
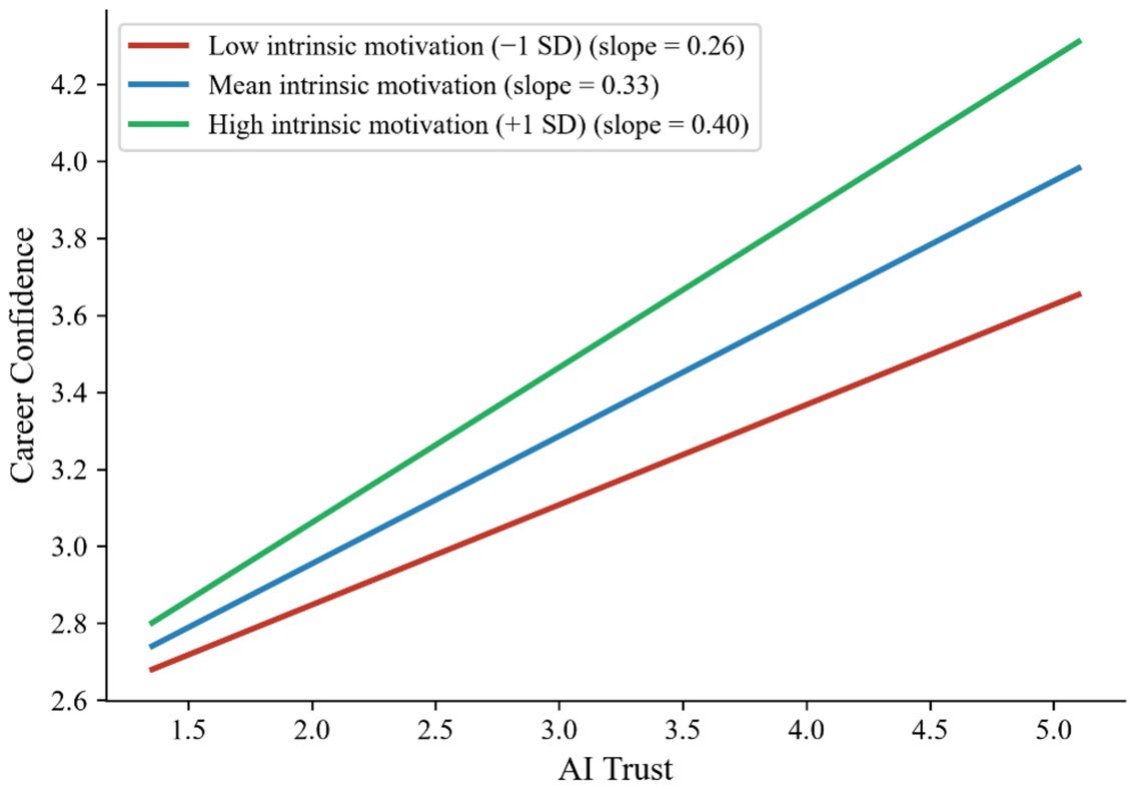
Simple slope analysis.

To further characterize the moderating effect, we conducted a Johnson–Neyman (JN) analysis. The simple slope of AI trust on career confidence was statistically significant for intrinsic motivation values of 2.10 or above (JN region: IM ≥ 2.10, i.e., approximately 2.2 standard deviations below the mean), which covers nearly the entire observed range of intrinsic motivation in the present sample (1.50–5.00). This indicates that the positive association between AI trust and career confidence was significant across the vast majority of the observed distribution of intrinsic motivation, with the exception of very low values (IM < 2.10). Figure 2 has been updated to report the simple slopes with their values, and the effect size of the interaction (ΔR^2^ = 0.006, f^2^ = 0.008) is reported in Section 5.1.

## 5 Discussion

### 5.1 Main findings

Performance expectancy was positively associated with AI trust, as was effort expectancy. This supports the key underpinnings of the UTAUT model (Venkatesh et al., 2003) and is in line with Yilmaz et al.'s (2024) empirical results in the context of generative AI. Consistent with the systematic review by Tamilmani et al. (2021), the effect of performance expectancy was larger than the effect of effort expectancy. This divergence is plausible for two reasons. Because generative AI communicates through natural language dialogue, its ease-of-use barriers are far lower than those of traditional systems, so students’ ease-of-use perceptions are fairly homogeneous and the actual functional benefit of AI becomes the main basis for differentiating trust. Moreover, amid the popularization of higher education and rising competition in China’s employment market, students care more about what AI can do for them than about the convenience of operation.

There was a positive relationship between AI trust and career confidence, as well as between career confidence and career goal setting. These findings position career confidence as the key connective node between technological attitudes and career behaviors. This finding offers direct evidence for the applicability of SCCT in the AI context. AI trust can serve as a technological form of environmental support and is positively associated with career confidence, which is in turn positively associated with career goal setting (Lent and Brown, 1996). The findings are in line with previous research on technological perceptions and career outcomes in organizational contexts by Kong et al. (2023) and Kong et al. (2021). They also support Jiang and Chen's (2026) finding that AI trust is associated with protean career orientation (Briscoe and Hall, 2006). In line with the present results, Testa et al. (2026) argued that AI literacy alone is not enough and needs to be translated into AI readiness and AI self-efficacy to improve career adaptability. Similarly, El-Sayed et al. (2025) discovered that AI literacy and innovative thinking significantly boost nursing students’ career confidence via career and talent self-efficacy.

Performance expectancy and effort expectancy each indirectly supported career goal setting via the serial pathway of AI trust and career confidence. However, a key conclusion can be drawn – once career confidence, trust in AI, effort expectancy, and performance expectancy were all included in the overall model (Model 6), the direct effect of performance expectancy was no longer significant (*b* = 0.051, ns), whereas that of effort expectancy, although still significant (*b* = 0.134, *p* < 0.05), was substantially attenuated relative to its total effect, while career confidence remained a strong predictor. Both serial mediation effects were significant, indicating that AI trust and career confidence jointly transmit the associations of performance expectancy and effort expectancy with career goal setting. This result provides strong quantitative evidence supporting the SCCT pathway, whereby technology acceptance antecedents are indirectly associated with career outcomes via career self-efficacy.

Intrinsic motivation was a positive moderator of the relationship between AI trust and career confidence. This is consistent with self-determination theory. Additionally, students who are more intrinsically motivated to apply generative AI to their career tend to reflect more on how generative AI can be incorporated into their career and are more inclined to act on the career insights they gain from generative AI, which strengthens the positive association between technological trust and career confidence. Moreover, this finding adds to Jiang and Chen (2026), who identified intrinsic motivation as a mediator of the relationship between AI trust and protean career orientation. The present study demonstrates that intrinsic motivation also conditions the strength of the association between AI trust and career confidence, suggesting that motivation plays a multiple role in the AI–career relationship.

Regarding the practical magnitude of this moderating effect, the interaction term explained an additional 0.6% of the variance in career confidence beyond the main effects (Δ*R*^2^ = 0.006, *f*^2^ = 0.008), which corresponds to a small effect size by conventional standards (Cohen, 1988). Accordingly, although the moderating effect is statistically significant, its substantive magnitude should be interpreted cautiously: the theoretical significance of this finding lies mainly in the consistent direction of the pattern—that is, the association between AI trust and career confidence was stronger at higher levels of intrinsic motivation—rather than in the magnitude of the difference.

### 5.2 Theoretical implications

First, this study achieves interdisciplinary integration of the UTAUT and SCCT frameworks by constructing a mechanism in which technology acceptance antecedents relate to career goal setting via AI trust and career confidence. To answer the long-standing call in the technology acceptance literature to move beyond the perspective of usage behaviors (Tamilmani et al., 2021), and focus on distal downstream consequences of technology attitudes, the outcome variables of UTAUT were extended in this integration, to include career psychological outcomes. Moreover, this research considers technological environmental perceptions (AI trust), as part of environmental supports in SCCT. It extends the number of antecedents included within SCCT and addresses the historical focus of the theory on social environmental supports (e.g., families, teachers, peers). The results can serve as a reference for further interdisciplinary studies to combine theories of technology and theories of career.

Second, by anchoring trust in SCCT as a pivotal node connecting the technological environment and career psychology, this study expands the theoretical relevance of SCCT in the digital career ecosystem. Most of the environmental factors discussed in the original SCCT are those offered by social factors like family, school, and economic situations (Lent et al., 1994; Lent and Brown, 1996), and few investigations have addressed the association between technological environments and career development. This study highlights that AI trust is a key link between technology acceptance and career confidence, showing that an individuals’ perception of AI technology, such as its reliability and usefulness, are crucial for moving from technology acceptance to career confidence. This integration of trust, a social psychological construct, in the environment–self-efficacy pathway of SCCT and helps expand theories related to psychological processes for how environment comes to be a reality. Based on a full assessment of technological competence, reliability and intentions, Glikson and Woolley (2020) proposed that technology trust would be built. The present study finds that such trust judgments are also positively associated with career self-efficacy and thus unveil the bridging link between technological perception and career psychology. These findings suggest that technology trust can become a novel source of environmental support along with family support and educational quality in the highly pervasive integration of AI into the career ecosystem.

Finally, by revealing the conditions under which the relationship between AI trust and career confidence operates, the moderating effect of intrinsic motivation provides a theoretical understanding of differential associations of technological empowerment by individual attributes. This study verifies that the relationship between AI trust and career confidence is positively moderated by intrinsic motivation. Behaviors that are intrinsically motivated are characterized by increased autonomy and persistence, according to SDT. The present study’s moderating findings suggest that the same applies to the process of technology trust to career psychology: AI interaction with high intrinsic value and interest identification intensifies this positive association. Moreover, this finding is consistent with the primary principle of Person–Environment Fit theory (Guan et al., 2021) that when technological environment (AI trust) and personal characteristics (intrinsic motivation) are more aligned with each other, the facilitation association with career development is stronger. It provides a clue that future studies should be done from technology–individual interaction perspective, not just have isolated main effects from the technical aspects.

### 5.3 Practical implications

The practical implications below are presented in four recommendations. Within each recommendation, we clearly distinguish the empirically supported core—which follows directly from the corresponding finding of this study—from the broader educational measures that additionally draw on the UTAUT and SCCT frameworks. Because the evidence comes from a cross-sectional survey, the latter measures should be regarded as suggestive guidance awaiting further verification.

First, directly supported by our finding that performance expectancy was more strongly associated with AI trust than effort expectancy, universities should emphasize the functional value of generative AI in education. Rather than limiting AI education to the popularization of AI concepts, universities should create teaching procedures based on hands-on experience and integrate AI tool training into profession-related subjects, so that students can directly experience the usefulness of AI. From a broader educational standpoint, the provision of unified campus AI platforms, operation training, and peer support communities can further reduce usage barriers and strengthen students’ effort expectancy, and teachers should avoid overhyping AI technologies to prevent unrealistic expectations—as sustainable trust needs to be built on consistent practical experiences (Glikson and Woolley, 2020). When users’ expectations of actual AI performance are not met, their trust can break down rather quickly.

Second, consistent with our finding that AI trust was positively associated with career confidence and career goal setting, universities may consider incorporating AI into career guidance, so that students can develop a cognitive model of human–AI collaboration and recognize career confidence as an important bridge between technological confidence and career readiness. More broadly, career guidance incorporating AI can be conducted at three levels. At the cognitive level, specialized workshops on AI and future jobs can help students grasp the potential of AI and its associations with job opportunities in different industries (Bankins et al., 2024), and reduce information asymmetries that can cause career anxiety. At the behavioral level, AI collaboration competencies can be integrated into career planning courses to develop the skills needed for job seeking and career exploration. At the psychological level, interventions can be designed to help students reframe AI from a threat to an enabler, thereby addressing career displacement anxiety; given that AI trust was positively associated with career confidence in this study, interventions aimed at fostering calibrated AI trust may therefore be worth further evaluation (Hirschi and Koen, 2021).

Third, directly supported by our finding that intrinsic motivation positively moderates the association between AI trust and career confidence, universities should establish tiered interventions that combine universal support with targeted enhancement. Because the positive association between AI trust and career confidence holds even for students with lower intrinsic motivation, AI trust per se provides a basic facilitation association that universal exposure to AI can leverage. Beyond this empirically grounded core, universities can provide platforms for autonomous exploration (e.g., advanced AI projects, innovation competitions, and interdisciplinary collaboration) for intrinsically motivated students to amplify the empowering association of AI trust; for students with low intrinsic motivation, structured AI courses may help students develop more informed perceptions of AI, and external rewards (e.g., credit recognition and project rewards) combined with meaning-making support can gradually shift extrinsic motivation toward autonomous motivation (Deci and Ryan, 2000).

Fourth, based on our finding that AI trust and career confidence serially mediate the associations of performance expectancy and effort expectancy with career goal setting, students should proactively create career preparation plans beginning with cultivating trust in AI. Taking a broader educational view, students can begin by using AI tools in authentic academic tasks to understand their possibilities firsthand, then intentionally connect AI use with their own career development goals—for example, using AI for career exploration, fine-tuning CVs, and gaining new skills—thereby turning trust in technology into career confidence and goal-setting behaviors. Students should also reflect on the motivation behind their AI use: by setting AI learning tasks that correspond to future career plans and engaging in AI innovation practices, externally required adoption can gradually evolve into intrinsically motivated exploration of AI, converting trust in technology into career-relevant psychological resources (Deci and Ryan, 2008). Given the fast evolution of AI technologies, students should adopt a growth mindset and adjust their perceptions of AI’s capabilities, creating a self-reinforcing cycle between technological capability and career confidence.

### 5.4 Limitations and future directions

First, we adopted a cross-sectional approach—all our variables were measured at a given point in time, so that it is not possible to draw causal conclusions between variables. While we proposed a theoretically ordered process model linking technology acceptance, AI trust, career confidence, and career goal setting, with a theoretical reasoning based on the UTAUT and SCCT theories, a cross-sectional study does not exclude the possibility of reverse causation. For example, it is equally plausible that career confidence precedes AI trust—that is, students who are more confident about their future careers may develop more favorable perceptions of AI—so that alternative models with a reversed ordering of these variables cannot be ruled out with the present design. The assumed temporal ordering is nonetheless grounded in the theoretical integration of UTAUT and SCCT, with UTAUT informing the positioning of technology–related perceptions as antecedent appraisals and SCCT providing the environment–self-efficacy–goal pathway for the subsequent career-related relationships (Lent et al., 1994). Longitudinal designs (e.g., three-wave time-lagged studies) and experimental designs (e.g., randomly assigning students to AI products of varying functionality) would help confirm causal directionality.

Second, our study has measurement-related limitations. On the one hand, we operationalized career preparation using only two of the eight CEDS subscales—career confidence (an attitudinal component) and career goal setting (a behavioral component). As noted in Section 2.1.3, career preparation is a multidimensional construct that encompasses additional attitudinal and knowledge-based components as well as further behavioral components beyond goal setting (e.g., career decision-making, information exploration, and engagement), so the associations of technology acceptance with these other components of career preparation may not be fully captured; future research could add more dimensions (e.g., decision-making and career information seeking) or broader constructs such as career adaptability as outcome variables. On the other hand, all variables were measured via student self-reports. Although the procedures reported in Section 4.1 indicate that common method bias did not materially affect our findings, limitations associated with a single data source remain: self-reported data are susceptible to social desirability and recall biases, and students may overestimate their positive attitudes toward AI. Future research could adopt multi-source data (e.g., peer ratings, instructor evaluations, objective usage logs) or combine quantitative findings with qualitative approaches (e.g., in-depth interviews and focus groups) to further strengthen the robustness and interpretive depth of the results. In addition, we acknowledge that the AI trust measure adopted here reflects a broader conception of trust than the narrow, dyadic trust examined in much of the trust literature: it encompasses both confidence in AI’s capabilities and positive expectations about the consequences of AI for work and careers. It may therefore partially overlap with adjacent constructs such as technology optimism or positive career expectations, and our findings should be read with this broader operationalization in mind; future work using measures that more narrowly target the interactional, dyadic aspects of trust would help delineate these boundaries.

Third, although the regression-based strategy adopted here is transparent and directly aligned with the hypothesized model, structural equation modeling (SEM) would represent an alternative and potentially advantageous analytical strategy for future research: by estimating the latent measurement and structural models simultaneously, it would account explicitly for measurement error and provide a more integrated test of the serial mediation and moderation. Future studies with larger samples could therefore complement the present findings with SEM.

Fourth, the set of variables included in our model remains limited. We examined intrinsic motivation as the sole moderator and only tested its moderating effect on the pathway from AI trust to career confidence, so the boundary conditions operating at other stages of the serial mediation model remain unclear; future research could investigate additional moderators (e.g., major type, AI anxiety, parental career expectations) and extend moderation analyses to other stages of the pathway. Moreover, several factors that could plausibly be associated with both AI trust and career outcomes—such as the frequency of AI use, prior experience with generative AI tools, AI competence or proficiency, and academic discipline (e.g., STEM versus non-STEM)—were not included as control variables, which may introduce omitted-variable bias; future research incorporating such variables (e.g., directly measuring AI usage frequency and AI literacy) would help rule out alternative explanations.

Finally, the sample consisted of undergraduate students predominantly located in Guangdong Province, China (66.4%), with a mean age of approximately 20 years. Although the respondents covered multiple disciplines and most provincial-level regions of China, the findings should be generalized with caution to students in other regions of China, to students in other countries, or to non-student populations (e.g., employed workers). Replication with more geographically balanced samples would further strengthen the external validity of our conclusions.

## 6 Conclusion

Drawing on an integrated theoretical framework combining UTAUT and SCCT, this study explores the mechanism through which generative AI acceptance is associated with college students’ career preparation using a sample of 539 college students in China. The main findings are as follows: (1) Both performance expectancy and effort expectancy are positively associated with AI trust, with performance expectancy showing a stronger association; (2) AI trust is positively associated with career confidence, which is further positively associated with career goal setting; (3) performance expectancy and effort expectancy are indirectly associated with career goal setting via the serial mediation pathway of AI trust and career confidence; (4) intrinsic motivation positively moderates the relationship between AI trust and career confidence. The conclusions offer a theoretical framework and empirical evidence for understanding college students’ career psychology in the AI era, and yield practical implications for universities to implement AI literacy education and career guidance services.

## Data Availability

The raw data supporting the conclusions of this article will be made available by the authors, without undue reservation.
