# Supplementary material for: The association between generative AI acceptance and college students’ career preparation: the serial mediating role of AI trust and career confidence and the moderating role of intrinsic motivation
Source: Front Psychol. 2026 Oct 8;17:1954368. doi: 10.3389/fpsyg.2026.1954368 (PMC13687052; doi:10.3389/fpsyg.2026.1954368)
Supplement: Supplementary file 1 [file Supplementary_file_1.docx]

**Appendix Measurement**

**Table A1. Measurement Items and Factor Loadings**

| Variables | Items | Code | Factor loading | Reference |
| --- | --- | --- | --- | --- |
| Performance expectancy | I find generative AI applications useful in my daily life. | PE1 | 0.78 | Yilmaz, F. G. K., Yilmaz, R., & Ceylan, M. (2024). Generative artificial intelligence acceptance scale: A validity and reliability study. International Journal of Human–Computer Interaction, 40(24), 8703–8715. https://doi.org/10.1080/10447318.2023.2288730 |
|  | The use of generative AI applications increase my chances of achieving the things that are important to me. | PE2 | 0.69 |  |
|  | Generative AI applications help me get things done faster. | PE3 | 0.71 |  |
|  | Using generative AI applications increase my productivity. | PE4 | 0.73 |  |
|  | The use of generative AI applications make my life easier. | PE5 | 0.77 |  |
|  | Generative AI applications are useful for my daily life. | PE6 | 0.71 |  |
|  | The use of generative AI applications increase my chances of solving the problems I face. | PE7 | 0.70 |  |
| Effort expectancy | Learning how to use generative AI applications is easy for me. | EE1 | 0.76 | Yilmaz, F. G. K., Yilmaz, R., & Ceylan, M. (2024). Generative artificial intelligence acceptance scale: A validity and reliability study. International Journal of Human–Computer Interaction, 40(24), 8703–8715. https://doi.org/10.1080/10447318.2023.2288730 |
|  | I think it is easy to leverage generative AI applications. | EE2 | 0.72 |  |
|  | Generative AI applications are easy to use. | EE3 | 0.73 |  |
|  | It is easy for me to become skilled in using generative AI applications. | EE4 | 0.79 |  |
|  | My interaction with generative AI applications is clear and understandable. | EE5 | 0.77 |  |
| AI trust | I have confidence in the use of AI technology | AT1 | 0.69 | Kong, H., Yin, Z., Baruch, Y., & Yuan, Y. (2023). The impact of trust in AI on career sustainability: The role of employee–AI collaboration and protean career orientation. Journal of Vocational Behavior, 146, 103928. https://doi.org/10.1016/j.jvb.2023.103928 |
|  | I believe AI technology can facilitate routine and trivial tasks through automation | AT2 | 0.64 |  |
|  | I believe AI technology can be used reliably and consistently without failing | AT3 | 0.42 |  |
|  | I believe AI technology will consistently operate, providing adequate and efficient results across a wide range of processes | AT4 | 0.72 |  |
|  | I believe AI adoption will result in the creation of new jobs | AT5 | 0.72 |  |
|  | I have a positive attitude towards the adoption of AI | AT6 | 0.76 |  |
|  | I believe AI technology can help develop new skills that will benefit my career development | AT7 | 0.73 |  |
|  | I have a positive attitude towards its impact on organizational business operations | AT8 | 0.70 |  |
|  | I believe AI will positively change the way people work within the organization | AT9 | 0.73 |  |
|  | I believe AI adoption won’t reduce the focus on human skills such as creative intellect in my future job | AT10 | 0.54 |  |
|  | I believe AI adoption will enhance the quality of my future work | AT11 | 0.77 |  |
| Intrinsic motivation | To overcome the uncertainty brought about by AI, I hope that my future work will provide me with the opportunity to increase my knowledge and abilities | IM1 | 0.70 | Liang, X., Guo, G., Shu, L., Gong, Q., & Luo, P. (2022). Investigating the double-edged sword effect of AI awareness on employee’s service innovative behavior. Tourism Management, 92, 104564. https://doi.org/10.1016/j.tourman.2022.104564 |
|  | To overcome the uncertainty of AI, I want to know how well I can perform in my future job | IM2 | 0.73 |  |
|  | Overcoming the uncertainty of AI in my future career brings me joy | IM3 | 0.77 |  |
|  | Overcoming the uncertainty brought about by AI helps me with self-expression | IM4 | 0.77 |  |
|  | Regardless of the outcome of overcoming the uncertainty that AI brings, I am content with gaining new experiences | IM5 | 0.73 |  |
|  | I’m happier when I can set goals for myself to overcome the uncertainty that AI brings in my future career | IM6 | 0.80 |  |
| Career confidence | I feel confident that I have a good idea of what career direction(s) or pathways I want to take | CC1 | 0.84 | McCowan, C., McIlveen, P., McLennan, B., Ho, P., Lê, Đ. A. K., & Tran, N. D. (2024). Career education and development scale for secondary and tertiary students in Vietnam. The Career Development Quarterly, 72(2), 158–171. https://doi.org/10.1002/cdq.12347 |
|  | I am confident I will get appropriate employment/further study opportunities upon graduation | CC2 | 0.80 |  |
|  | I am confident I will have a successful future | CC3 | 0.84 |  |
| Career goal setting | I have set myself clear and achievable career goals | CGS1 | 0.79 | McCowan, C., McIlveen, P., McLennan, B., Ho, P., Lê, Đ. A. K., & Tran, N. D. (2024). Career education and development scale for secondary and tertiary students in Vietnam. The Career Development Quarterly, 72(2), 158–171. https://doi.org/10.1002/cdq.12347 |
|  | I have developed a career plan for myself | CGS2 | 0.83 |  |
|  | My career plans contain short, medium, and long-term goals | CGS3 | 0.81 |  |
